# A Newly Characterized, Two BRCT Domain-Containing Isoform of PAX-Interacting Protein (PTIP) Generated via Frame Shift and Alternative Pre-mRNA Splicing

**DOI:** 10.33696/signaling.6.143

**Published:** 2025

**Authors:** Ching-Jung Huang, Chuan Li, Danyang Yu, Hyein Cho, Kangsan Kim, Y. Jessie Zhang, Daechan Park, Haley O. Tucker

**Affiliations:** 1 Department of Biology, Arts and Sciences, New York University in Shanghai, Shanghai 200122, China; 2 Molecular Biosciences, University of Texas at Austin, 1 University Station A5000, Austin, TX 78712, USA; 3 Department of Molecular Science and Technology, Advanced College of Bio-Convergence Engineering, Ajou University 206 Worldcup-ro, Yeongtong-gu, Suwon 16499, South Korea

**Keywords:** B cells, Breast cancer C-Terminal, Lymphocytes, Plasma cells

## Abstract

In an effort to clone polyglutamine-rich factors from activated B lymphocytes of mice, we discovered and describe here a previously uncharacterized isoform of PTIP/PAXIP1. By virtue of a two-nucleotide frameshift followed by alternative pre-mRNA splicing, this shorter isoform of 576 amino acids (termed PTIP576) retained only the two central BRCT domains of previously characterized PTIP and encodes a unique and structurally disordered 50 residue C-terminus. PTIP576 is expressed primarily in nuclei of progenitor and activated mature B lymphocytes. Transgenic overexpression of PTIP576 in murine B and T cells led to increased lineages of bone marrow B cells and thymocyte CD4 T cells. We conclude by addressing potential functions of PTIP576 resulting from the relatively unique mechanism by which it is engendered.

## Introduction

Dressler and his colleagues [1] first discovered and characterized PTIP/PAXIP1 as a 1056 amino acid transcription factor (hereafter termed PTIP to distinguish it from PTIP576). The most notable feature of PTIP is its tandem array of 6 BReast cancer C-Terminal (BRCT) domains. BRCT domains are typically involved in DNA damage response, cell cycle control and transcriptional regulation via complexing with histone methyltransferases [2,3].

While PTIP is expressed ubiquitously [1] it was shown to engender the above described functions in the immune system via regulation of Variable-Diversity-Joining (VDJ) and Class Switch Recombination (CSR) recombination—processes inherent to differentiating B and T lymphocytes [4–12]. When B cells encounter their specific antigen, the B-cell receptor (BCR) and the subsequent initiation of downstream signaling pathways are activated (reviewed in [11–13]). Recognition of specific antigen, along with signals from interleukins (IL; including IL-2, IL-5 and IL-7) act as triggers for induction of “germline” antigen receptor transcription—a forerunner and requirement for induction of VDJ and CSR assembly. IL-2 also is crucial for inducing the differentiation of B cells into plasma cells which produce antibodies.

Another mechanism critical to the current investigation is frameshift mutation (reviewed in [14]). Formally this occurs when one or two bases is added or deleted, thereby disrupting the triplet codon, and thus, the reading frame of the mRNA. This shift in reading frame can lead to a completely different, but more likely, dysfunctional protein. Frameshift mutations near a pre-mRNA splice site can determine whether the splice site is properly recognized and processed. For example, and observed in the analysis to follow, the frameshift mutation in an acceptor splice site of an intron can lead to the inclusion of intronic DNA into the mature mRNA. They often have severe consequences for protein truncation, misfolding which underlay a number of genetic disease disorders (re- addressed in Discussion).

We recently demonstrated that, in addition to its activities reviewed above, PTIP catalyzes apoptosis via a standard mitochondrial-based mechanism [15]. While both PTIP and PTIP576 share considerable sequence identity, including several domains critical to PTIP’s apoptotic function (further detailed in Discussion), PTIP576 does not catalyze apoptosis (data not shown). In this report, we demonstrate that this previously uncharacterized isoform is expressed exclusively in hematopoietic linages and is generated from the same *Ptip/PaxIp1* locus via frameshift and alternatively pre-mRNA splicing, resulting in a 50 amino acid, novel C-terminus. Hematopoietic-specific transgenic overexpression of PTIP576 suggest roles both in progenitor and mature B cells as well as in mature T cells. We end with speculations regarding the mechanism by which this novel isoform might act.

## Results

### Cloning and initial characterization of PTIP576

In search of QR-containing factors, PTIP576 was cloned from an Interleukin (IL)-2+IL-5-induced LambdaZap library constructed from a murine B cell leukemia (Bcl1) cDNA library as described in Herrscher *et al.* [16]. We used as a probe, a fragment of IL-2 cDNA which contains 12 glutamine (CAG) repeats (details in Methods). As shown in Supplementary Figure 1, its cDNA is composed of 2,873 nucleotides predicting a transcript size of ~3.4 kb and encoding an open reading frame of 576 amino acids. Its predicted ~65 kD protein starts at the first AUG (nucleotides 324–326) which are not preceded by in-frame stop codons within a 323 nucleotide 5’ untranslated region. Its polyadenylation site (AATAAA) is located 12 nucleotides upstream of the poly(A) tail within an 819-base 3’ untranslated region; Genbank accession number: PV904237).

**Figure 1.**
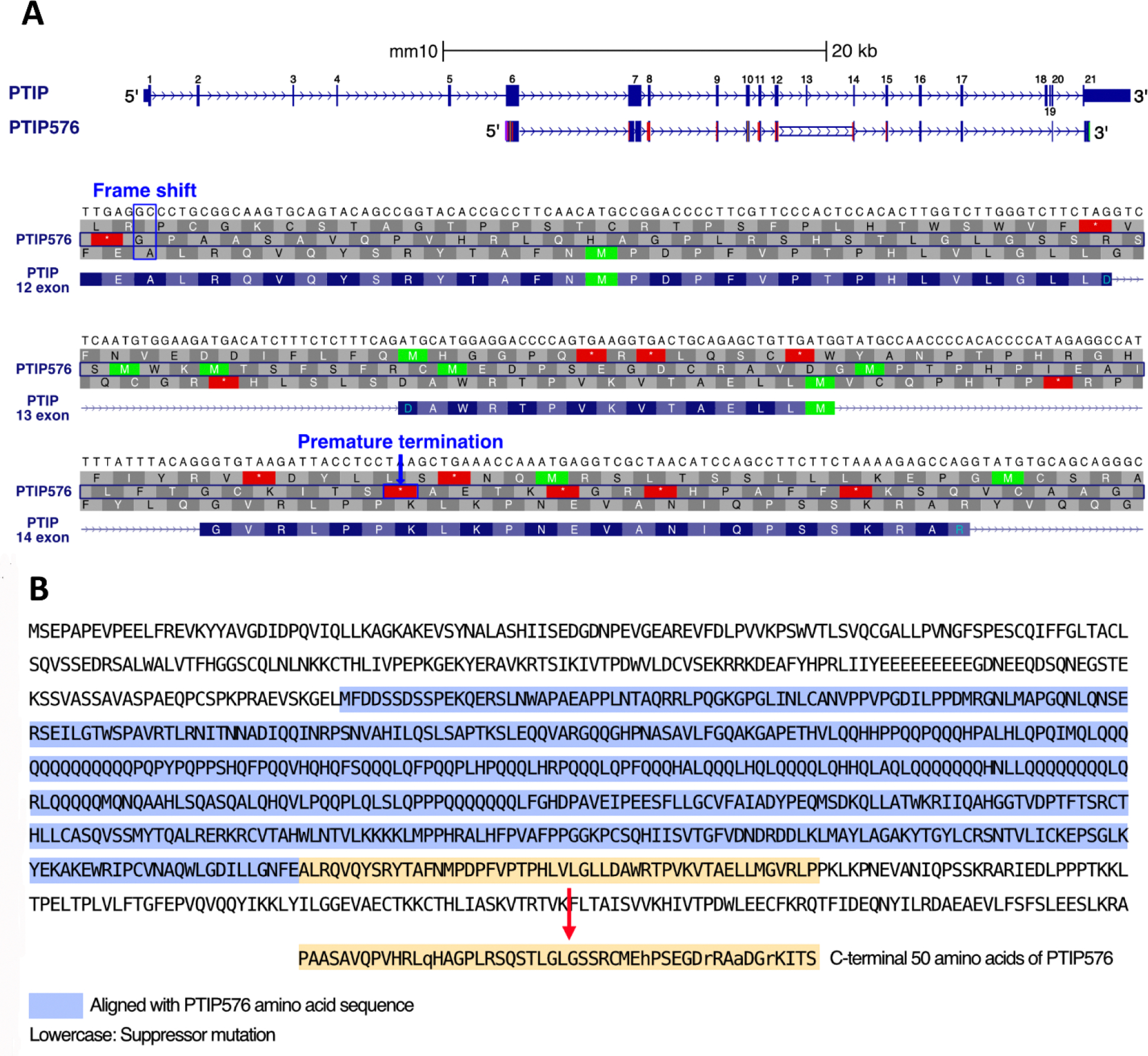
Comparison of the *Paxip1* gene and its 2 alternative PTIPs. (**A**) The DNA and amino acid sequences of PTIP and PTIP576 aligned with the exons of *Paxip1* [PMID: 39460617]. PTIP contains exons 1 through 21, whereas PTIP576 includes only exons 6, 7, 8, 9, 10, 11, 12, 14, 15, 16, 17, 20, and 21. The amino acid sequence of PTIP576 aligns with that of exons 12–14. However, in PTIP576, a two-base frameshift mutation occurs in exon 12, resulting in an altered amino acid sequence in exons 12–14 compared to PTIP which results in premature termination in exon 14. (**B**) Comparison of amino acid sequences of PTIP and PTIP576. The amino acid sequence (uncolored) corresponds to PTIP. The blue region represents the aligned amino acid sequences between PTIP576 and PTIP, while the yellow region indicates the unmatched C-terminal 50 amino acids of PTIP576. The lowercase letters indicate the amino acids that are altered from those encoded by the frameshifted *PTIP* gene, potentially resulting from a subsequent, intragenic suppressor mutation in PTIP576. This appears to correct the codon so that it can restore the reading frame that was disrupted by the frameshift.

That the ORF of PTIP576 than is considerably shorter than of PTIP led to our initial suspicion that PTIP576 might encode a previously uncharacterized isoform. Further shared with PTIP are a highly acidic region (36%) composing amino acid residues 1–14 and a prototypic nuclear localization signal (NLS, residues 412–430) (Supplementary Figure 1). A second notable feature of both PTIPs is a highly glutamine rich (QR) region spanning amino acids 155–333 of PTIP576 and 397–577 of PTIP (Supplementary Figure 2). These regions are >63% glutamines with several long polyglutamine repeats of uninterrupted lengths varying from 6 to 13 (Figure 1A).

However, and as shown in Supplementary Figure 2, while PTIP contains 6 BRCT domains, PTIP576 only has the central two, BRCT3 and 4, with respect to the nomenclature previously established for PTIP by Lechner *et al.* [1]. More notably, PTIP576 encodes a unique, previously unidentified C-terminal 50 amino acid stretch termed E12–14 and described below.

### The C-terminal 50 amino acids of PTIP576 are generated by frame shift and alternative pre-mRNA splicing

The *PTIP/PAXIP1 interacting protein 1* locus is composed of 21 exons on chromosome 5 [17]. The full-length cDNA of PTIP576 is encoded from exons 6–17, 20, and 21 (Figure 1A). The alignment between mPTIP1576 and PTIP indicated that the identity between the two isoforms lies primarily within the central region of PTIP, which includes the BRCT 3 and 4 domains of PTIP (Supplementary Figure 2).

Their alignment further indicated that PTIP576 is an alternatively pre-mRNA spliced isoform of mPTIP1576 (Figure 1B). Further the alignments indicated that the C-terminal 50 amino acids of PTIP576 (Figure1B; Supplementary Figure 1) aligned neither with any sequences within PTIP nor within any sequences in the NCBI database. When examined closely, these 50 amino acids were assembled by three tracks of peptide fragments because of frame shifts of Exon 12, 13, and 14 (Figures 1A and 1B).

**Figure 2.**
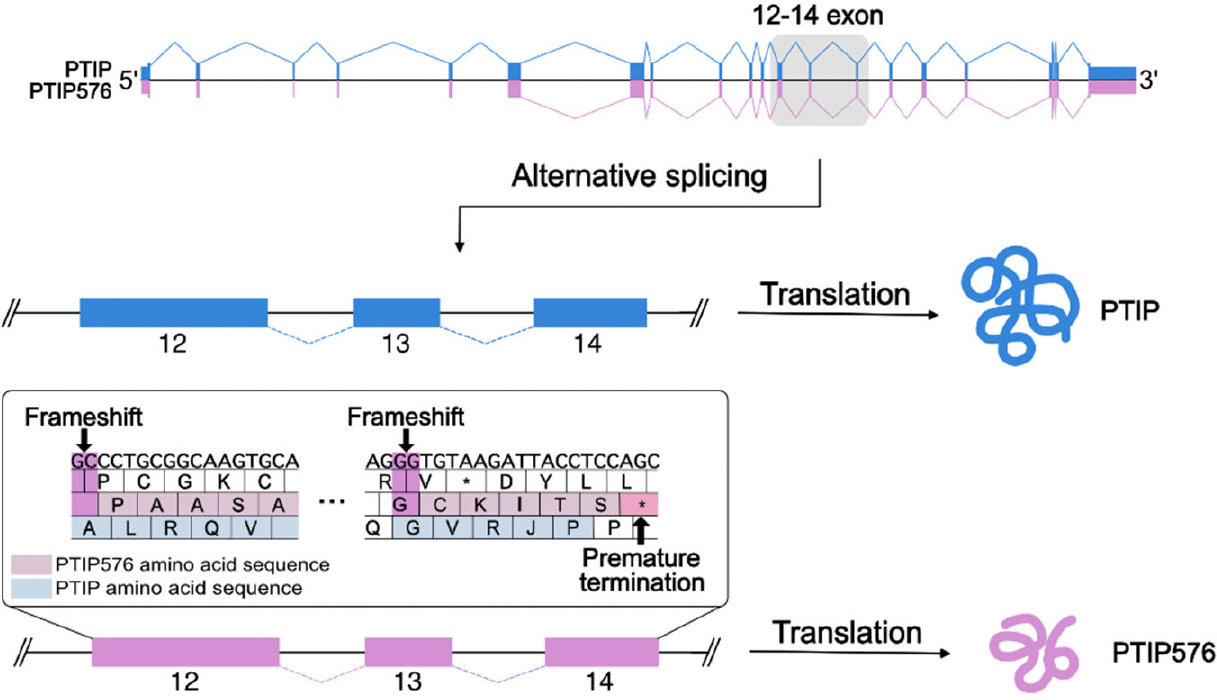
Cartoon illustrating the frame shift and alternative pre-mRNA splicing that generates PTIP and its isoform PTIP576. The amino acid sequence shown represents the C-terminal 50 amino acids of PTIP576 that are altered compared to those of PTIP due to a two-base frameshift mutation. These amino acids are encoded by exon 12 (position 1–30), exon 13 (position 31–44), and exon 14 (position 45–50). The lowercase letters indicate the amino acids that are altered from those encoded by the frameshifted PTIP DNA due to a subsequent suppressor mutation in PTIP576.

The detailed sequences and frame shifts are summarized in Figure 2. The first 30 amino acids encoded by this terminal three exon-derived E12–14 domain are readdressed in Discussion.

### PTIP576 expression is developmental stage-restricted within the B cell lineage

**Figure 3.**
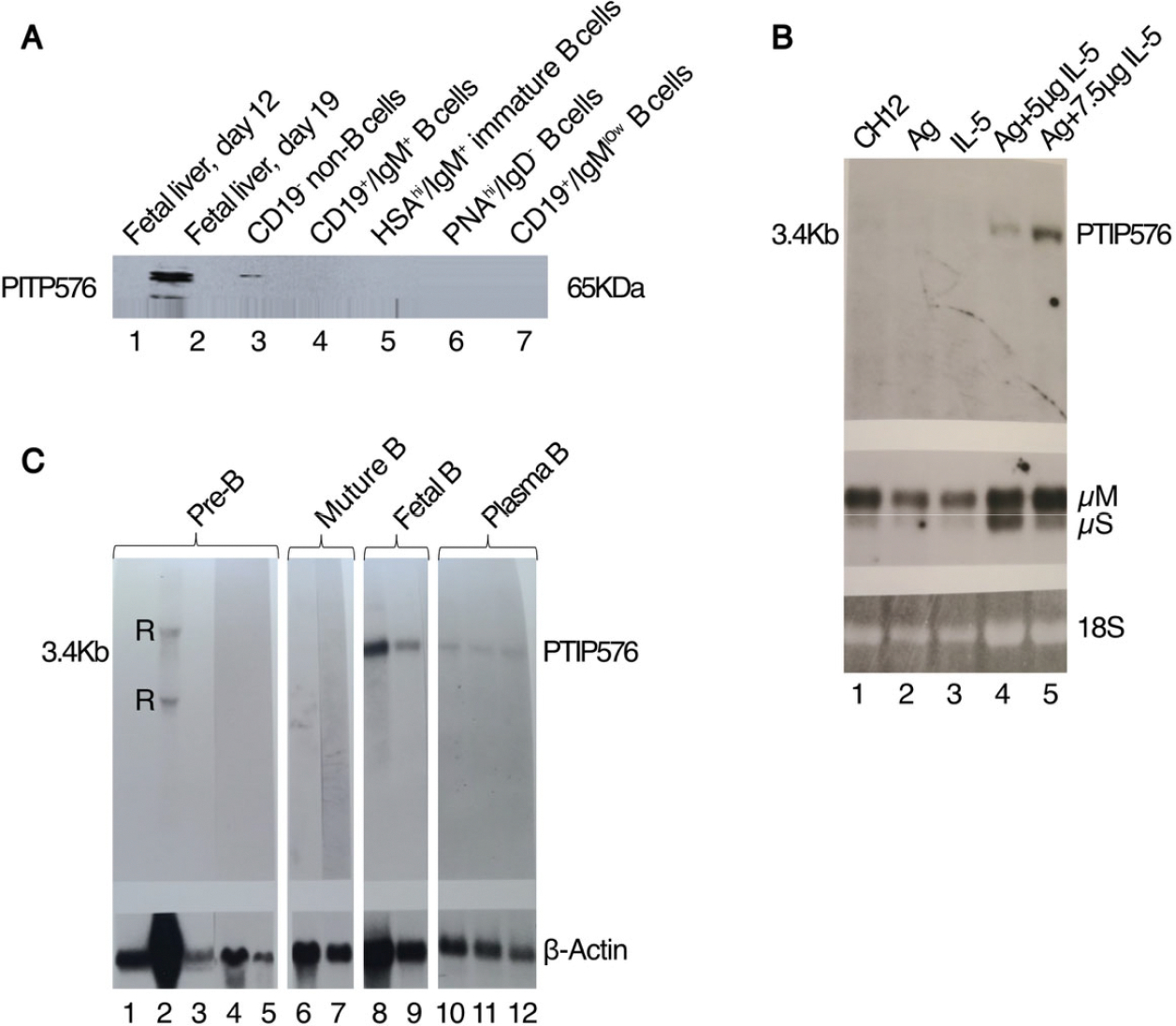
PTIP576 expression is developmental stage-restricted within the B cell lineage. (**A**) Expression of endogenous PTIP576 during mouse embryonic development in fetal liver. ~20 μg of cell lysate from each cell population (indicated above) was fractionated on 10% SDS-PAGE, transferred to nitrocellulose and then probed with a conventional mouse monoclonal antibody against PTIP (BRL Gibco). While the Ab should pick up both conventional PTIP and PTIP576, conventional PTIP is not expressed in hematopoietic progenitors. The antigen/antibody complexes were visualized by ECL. PTIP576 expression was restricted to unfractionated E19 fetal liver and to E19 fetal CD19+IgM+ B cells. (**B**) Northern analysis reveals PTIP576 mRNA is induced with antigen (AG) and Interleukin-5 (IL-5) in CH12 murine B cells. Inductions were performed employing phosphorylcholine (PC) as antigen (Ag) coupled to KLH and recombinant IL-5 as previously described [42]. Upper panel: Lane 1, CH12 alone; lane 2, Ag alone; lane 3, IL-5 alone; lane 4, Ag + 5 μg IL-5; lane 5, Ag + 7.5 μg IL-5. Center panel: Membrane and secreted forms of IgM (μS and μM) hybridizations; lower panel:18S rRNA loading controls. (**C**) Northern blot analysis reveals PTIP576 mRNA accumulates primarily in adult mature B cells lineages. Lane 1, neonatal splenic B cells; lane 2, long term cultured, IgM negative bone marrow-derived normal pre-B lymphocytes; lane 3, 70Z/3, lymphoid bone marrow cells cultured in the presence (Lane 4) and absence (lane 5) of IL-7; lanes 6 and 7, Bcl-1 and M12 murine leukemic mature B cells; lanes 8 and 9, IgM-secreting hybridomas, 29L4 and 29L6 derived from fetal liver; lanes 10–12, IgM-secreting hybridoma fusion of Bcl-1 and X63. β-actin hybridization of these RNAs is indicated in the lower panel. Cross hybridizations against ribosome RNA (r) are indicated.

PTIP was shown to be expressed ubiquitously, reaching high to moderate levels in both fetal and adult compartments [1]. However, as shown in the Western analysis of Figure 3A, expression of endogenous PTIP576 was detected in fetal liver E19 CD19+IgM+ B lymphocytes, but not in E12, nor in CD19^−^ non-B cells, HSA^hi^/IgM^+^ immature B cells, nor PNA^hi^/IgD^−^ germinal center B cells.

In normal adult hematopoietic lineages, expression of PTIP576 is extremely modest and only barely detectable by Western blotting in the adult mouse with the exception of low levels in testis, liver, brain and thymus (Supplementary Figure 3).

However, since PTIP576 was cloned from an IL-2 + IL-5-stimulated BCL1 mature B cell leukemia library [16], we employed an inducible cytokine and antigen (Ag)--specific approach [16]. BCL1 was first stably transfected with Cμ and Vκ genomic V regions (termed V_H_S107) that conferred the T15 Idiotype and phosphocholine (PC)-binding specificity upon the cells [18]. Next, the transfected cells were treated with both the T-dependent Ag [phosphorylcholine-key-hole limpet hemocyanin (PC-KLH)], and 0.5 ng/ml purified IL-5. As shown in the Northern analyses of Figure 3B, after 3.5 days steady state PTIP576 mRNA levels increased dramatically, and the control Cμ levels increased three- to four-fold over levels obtained from untreated cultures, or cultures treated with IL-5 or PC-KLH alone [19]. IL-5 alone caused increased Ig secretion and a nearly two-fold increase in proliferation of PTIP576 transductants (data not shown). These data provided direct proof that PTIP576 transcription are highly inducible in B lymphocytes.

Further Western analyses implicated higher expression of PTIP576 RNA within fetal and mature B cell stages, whereas no signals were obtained from neonatal splenocytes, BM-derived normal pre-B cells (Figure 3C), nor from various B cell tumors or plasma cell hybridomas (data not shown).

Thus, unlike PTIP which is expressed at relatively high levels and ubiquitously throughout fetal and mature compartments [1], PTIP576 is restricted primarily to fetal and mature B cells. Perhaps its restricted expression pattern encumbered discovery of this isoform until ~30 years post characterization of PTIP.

### Generation and analysis of PTIP576 transgenic mice

HA-tagged PTIP576 was subcloned into pEmb-1, in which expression is controlled by a (reportedly) B cell-specific (mb-1 promoter) and the Eμ enhancer [20]. Purified and linearized HA-PTIP576 cDNA was subsequently injected into male pronuclei of fertilized C57Bl mouse eggs and the injected eggs were implanted into oviducts of pseudopregnant foster mothers. Dot blots were performed to screen the transgene from the born pups (Materials and Methods).

Two lines of founder mice were identified (termed 35–4 and 34–5) containing ~5 and ~20 copies, respectively. Anti-HA Western blots detected HA-PTIP576 in spleen and thymus, but not in liver nor in other tissues tested (Supplementary Figure 4; data not shown). Although the Eμ enhancer has been shown to drive expression in T cells [21], thymic expression was unanticipated given previous data indicating B cell specificity of the mb-1 promoter [22]. Nevertheless, it allowed us to analyze transgenic overexpression of PTIP576 in mature thymocytes as well as in early hematopoietic development. Both 35–4 and 34–5 transgenic mice were crossbred to generate transgenic homozygous alleles, but most of the preliminary data that follow come from the higher copy number, 34–5 line.

**Figure 4.**
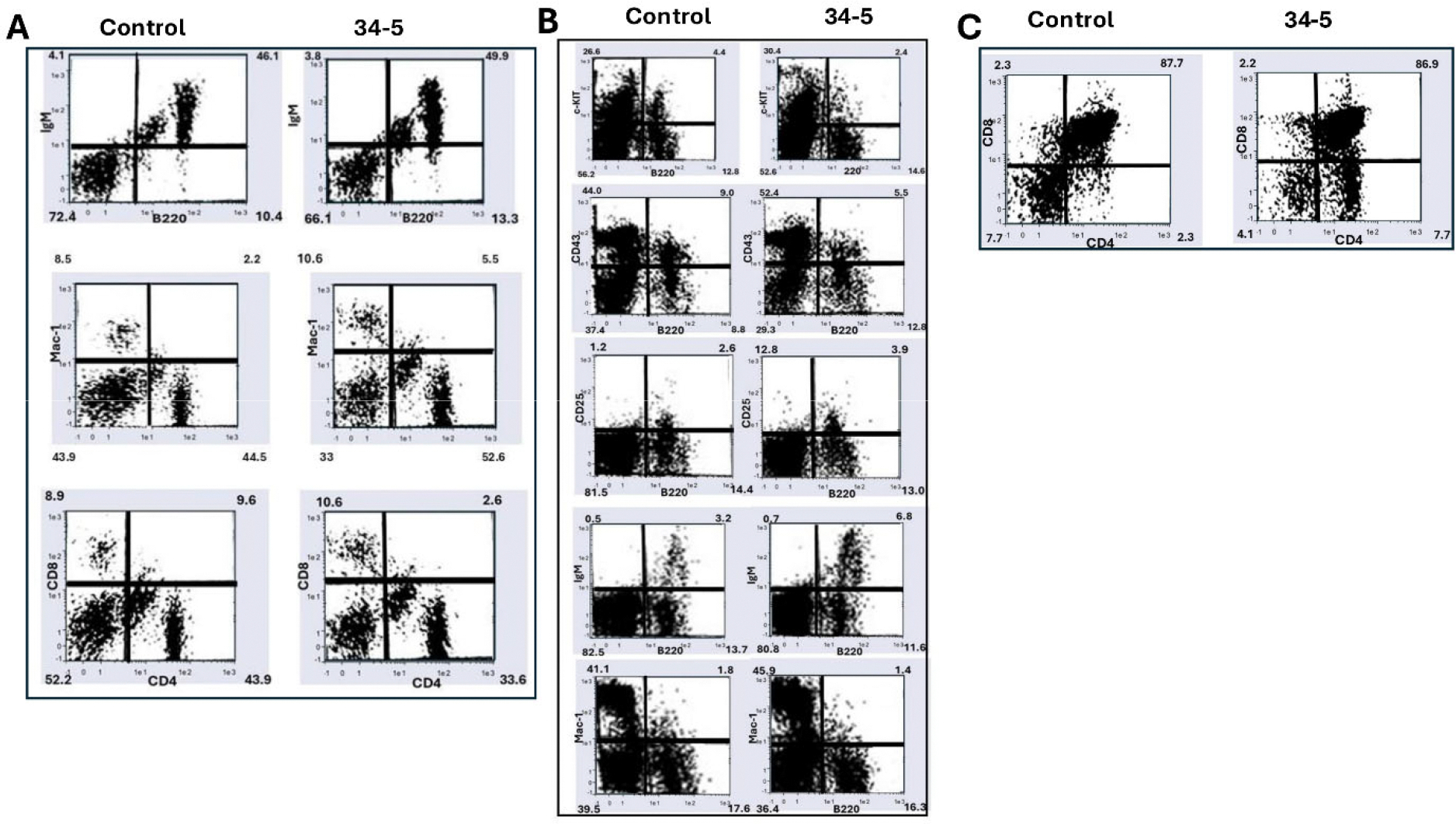
Flow cytometric analysis of murine hematopoietic cell lineages alterations following overexpression of PTIP576 in C57Bl/6 transgenic mice. (**A**) PTIP576 overexpression has no effect on peripheral lymphocyte populations in the spleen. Lymphocytes were isolated from spleens of controls (Ctr) and one of two strains (transgenic mice then were stained with the indicated antibodies (Abs) as detailed in Materials and Methods. Percentages of each population were determined by FACS analyses. Data shown are representative of 3 independent experiments (**B**) PTIP576 transgenic mice lymphocytes are deficient in pre-B cells but not in mature B cells. Both B220+cKit+ pre-B 1 progenitors as well as CD43+/B220 large pre-BIII are significantly reduced (p≤0.05; n=4). Conversely significant (p≤0.05; n=4) increases CD25+B220+ immature and in IgM+/B220+ mature (from 3.2 to 6.8%) populations are observed. PTIP576 overexpression results in no significant changes in total cell numbers nor in myeloid (Mac1+) nor mature CD4+ or CD8+ mature splenic T cells. (**C**) PTIP576 transgenic thymuses accumulate increased numbers of CD4 SP thymocytes. There are no significant alterations in CD8+ SP or CD8+CD4+ DP cells thymocytes, whereas CD4 SP thymocytes were increased ~3-fold increase (p≤0.01; n=4) relative to littermate controls (2.3% to 6.7%). This led to an ~2-fold increase in total thymocyte numbers (p≤0.01; n=4). The flow analyses shown and their data tabulation in Table 1 were representative of three independent determinations.

To examine the effects of PTIP576 expression on the peripheral B cell population, flow cytometric analysis initially was performed on splenic cells from mature (~6 weeks old) mice. As summarized in Table 1 and shown in Figure 4A, we observed no significant change in the percentages of B220+/IgM+ mature C cells, nor of Mac-1+ myeloid cells, nor of CD4+ or CD8+ single positive (SP) T cell populations. This indicated that over-expression of PTIP576 had no effect on mature lymphocyte accumulation. However, while we observed no obvious differences in the sizes of spleens or thymuses, the total numbers of transgenic splenocytes increased from ~8.9 X10^7^ to ~1.4 X 10^8^; a significant (p≤0.05; n=4) increase of 57±6.6%. This suggested that an alteration in B cell development had occurred, perhaps at an earlier stage.

### PTIP576 transgenic mice suffer early pre-B cell loss which is rescued in mature B cell lineages

To determine the stage at which B cell development was altered in PTIP576 transgenic mice, bone marrow (BM) from transgenic and non-transgenic mice were examined for expression of c-kit, CD43, CD25 and IgM—surface markers conventionally employed as markers of B cell developmental stages. For example, the expression of c-kit extends from progenitor cells to pre-BIII cells; CD43 is expressed from progenitor cells to large preB-II cells; CD25 is expressed from large pre-BIII cells to immature B cells; and IgM is only expressed on immature and mature B cells.

As summarized in Table 1 from the data of Figure 4B, the percentage of B220+cKit+ B cells, which represent the B cell population from progenitor to pre-B1, decreased from ~4.4% in nontransgenics to ~2.4% in transgenic mice—a significant reduction (p≤0.05; n=4) of ~45%. The percentage of CD43+/B220+ cells, which correspond to the B cell population from progenitor to large pre-BII cells, dropped from ~9.5% to ~5.5%—a significant (p≤0.05; n=4) decrease of 43% in transgenics.

These data suggested that expression of PTIP576 induced a block at the pre-BIII stage but was rescued in later B cell stages as evidenced by significant (p≤0.05; n=4) gains in CD25+B220+ immature (from ~2.6 to ~3.9%) and in IgM+/B220+ mature (from ~3.2 to ~6.8%) populations. These pre-BIII and mature B cell gains represented increases of ~50% and ~113%, respectively. As controls, we observed no significant changes in total cell numbers, neither in mature myeloid (Mac1+) nor mature CD4+ or CD8+ single positive (SP) splenic T cells (Table 1).

### PTIP576 transgenic mice are deficient in CD4 SP thymocytes

The unexpected expression of the PTIP576 transgene in the thymus prompted us to investigate the effect of its overexpression on T cell development. Thymocytes from transgenic and nontransgenic controls were analyzed by flow cytometry (details in Materials and Methods). As summarized in Table 1 and shown in Figure 4C, while no significant changes from control thymuses were observed in CD8+ SP or in CD8+CD4+ double positive (DP) cells, an almost 3-fold increase of CD4+ SP cells (**p≤0.01**; n=4) was observed in PTIP576 transgenic mice relative to littermate controls (~2.3% to ~6.7%).

This strongly suggested that PTIP576 expression promotes uncommitted DP T cells to develop into CD4+ cells. Consistent with that observation, total thymus cell number isolated from PTIP576 thymuses increased from ~6.2 × 10^7^ to ~1.3 × 10^8^; an increase by 110% (p≤0.01; n=4).

Hypotheses to explain these unexpected results are provided below in Discussion.

## Discussion

### Frame shift and alternative pre-mRNA splicing within exons 12, 13, and 14 of the *PAXIP1* (PAX Interacting Protein 1) gene locus created the unique C-terminus of PTIP576

The amino acid sequence of PTIP contains 21 exons. However, the open reading frame of PTIP576 initiates within exon 6 and extends through exon 17 and includes only exons 20 and 21. Unexpectedly, three reading frame shifts occur at exons 12, 13, and 14 which generate the unique isoform PTIP576. This results in inclusion of only BRCT 3 and 4 of PTIP and a unique C-terminal 50 amino acids that we termed E12–14 (Figure 1B). This alternative splicing/frame-shift mechanism generated a novel, previously uncharacterized C-terminal domain whose function is hypothetically considered at the end of the Discussion.

This unusual mechanism (illustrated in Figures 1A and 1B) appears to be attributed to deletion of two bases “GC” within exon-12. Comparing the sequences of PTIP and PTIP576, this “GC” resides within exon-12 but not adjacent to the intron. The “TTGA” in exon-12 just upstream of “GC” is also retained to encode the Phe-Glu residues shared by PTIP and PTIP576. We suggest that this cannot directly be mediated by alternative splicing, but rather a deletion mutation of the ‘GC’ nucleotides at the genomic DNA level.

There are several genetic diseases that are caused by frameshift mutations, including Tay-Sachs Disease, Cystic Fibrosis, Crohn’s, Charcot-Marie-Tooth Disease (Hereditary Polyneuropathy), and Hypertrophic Cardiomyopathy; a specific frameshift mutation in the *CCR5* gene has also been linked to HIV resistance (reviewed in [14,23,24]). Most, if not all of these lead to loss of function. Another class of frameshifts, termed Extended Incorrect Terminus variants (EITs), which may include PTIP576, also have been observed in some disease-associated genes, and often result in change of function. For example, in the case of the *NR3C2* gene, the frame shift creates a condition of salt wasting and resistance to mineralocorticoids [25]. Alternatively, in the Wilms Tumor 1 (*WT1*) gene, EIT leads to unbridled cell proliferation [26,27]. Of further relevance to the present situation, six BRCA1 frameshift variants have been described and functionally assessed [28,29]. Only one of these leads to an extended EIT, whereas the other five result in premature protein termination, and importantly, all displayed loss of function.

### Preferential expression of PTIP576 within B cell hematopoietic lineages

We demonstrated that, unlike the ubiquitously expressed PTIP isoform, PTIP576 is expressed predominantly within the B linage—both from cells isolated at embryonic (E) days E12 and E15 via timed pregnancies as well as from cell lines derived at various stages of adult B cell differentiation.

During murine embryonic development, the fetal liver is the primary site of B cell development prior to the bone marrow (BM) taking over after birth. At E12.5 onwards, the fetal liver becomes the critical organ for both B lymphopoiesis and myelopoiesis with progenitors and precursors developing progressively over time. By E12.5, IgH chain-expressing cells, negative for PTIP576, can be detected in the fetal liver [19]. However, PTIP576 expression is first detected exclusively in E19 CD19+IgM+ B progenitors, but not in progenitors of later lineages, including CD19^−^ non-B cells, HSA^hi^/IgM^+^ immature B cells, nor PNA^hi^/IgD^−^ germinal center BM progenitors (Figure 3A).

While more definitive data is required from normal peripheral B cells, model pre-B and immature transformed B cell lines were devoid of PTIP576 mRNA expression (Figure 3C). Yet compelling evidence for reemergence of PTIP576 expression was provided by its impressive Ag + IL-5 induction Ag-specific BCL1 mature B cells (Figure 3B).

Taken together, we speculate that, relative to the ubiquitously expressed PTIP, the PTIP576 isoform is B cell-restricted due to its unique 5-untranslated and/or E12–14 C-terminus.

### Comparable results of PTIP576 overexpression and PTIP knockout in B cells

Given that PTIP576 was most highly expressed in the B cell lineage (Figure 2), we utilized a B cell-specific promoter (*mb-1*) and enhancer (*Eμ*) cassette to drive overexpression of HA-tagged PTIP576 in C57A mice. As shown in Figure 4 and summarized in Table 1, PTIP576 overexpression led to a decrease in B cell progenitors at early developmental stages, yet an increase in mature B cells which accumulate at late stages of development. With respect to previous knockout results of conventional PTIP [1,11–13], we observed primarily reciprocal results.

Conventional PTIP KO leads to partial blocks in B cell development and profound immunodeficiency [30]. The B cell compartments of both PTIP mutant strains showed differences from controls in pre-pro-B numbers, whereas pro-B levels were significantly increased in the knockout and reduced in transgenic BM (Figure 4; Table 1). Relative numbers of small pre-B decreased ~2-fold in PTIP KO BM, while they were increased to a comparable level in transgenics. Numbers of large pre-B cells and mature B cells in BM were concomitantly increased in the knockout, but as expected, were down in the transgenics comparable to controls (Figure 4B; Table 1).

From these results, it was concluded [28] that loss of PTIP576 led to a mild B cell development defect marked by an inefficient transition of large cycling pre-B cells to small quiescent pre-B cells. Consistent with this conclusion, increased transgenic expression of PTIP576 led to the opposite effect at a developmental timepoint in which Ig light chains are primed to undergo rearrangement, once the pre-B receptor is engaged prior to BCR signaling.

However, the above-mentioned similarities fail to explain the increase in mature B cell percentages at late developmental stages in PTIP576 transgenics. Perhaps the expression of exogenous PTIP576 in B cells drives more B cells at early developmental stages into late stages. Regardless and unlike PTIP, it appears that exogenous expression of PTIP576 does not delete B cells through apoptotic effects demonstrated in PTIP-overexpressing cell lines [15]. This is consistent with our observation that no significant change in total numbers of bone marrow or splenocytes were observed (Table 1).

Western blot of tissue lysates revealed that the expression level of PTIP576 in spleen and liver was quite modest compared with the level of endogenous PTIP576 ([1]; Supplementary Figure 3). This might provide an alternative explanation as to why its transgenic overexpression did not induce detectable apoptosis in B cells. Trivial reasons for this observation might include instability of the epitoped transgene protein, which is consistent with the observation that expression levels of PTIP576 are far lower than those of PTIP in transiently transfected cells (data not shown). Furthermore, the transgene we generated lacks the normal 5’ UTR of genomic PTIP576.

In the mouse, B cells are generated from pluripotent hematopoietic stem cells in the liver during mid-to-late fetal development and in the bone marrow after birth. There are differences between hematopoietic development in fetal liver and adult bone marrow. Laky *et al.* [31] showed that in PTIP KO mice, B cell development in bone marrow proceeded to the B220^+^ CD43^+^ stage with normal levels of D_H_ to J_H_ rearrangement, although V_H_ to D_H_J_H_ rearrangement was severely repressed. In contrast, B lymphoid precursors were undetectable in fetal liver. The mechanism underlying the difference remains unknown.

### The unanticipated transgenic PTIP576 expression in the thymus specifically targeted CD4 SP cells

Given the published B cell specificity of the *mb-1* promoter/*Eμ* enhancer cassette [20], transgenic expression of PTIP576 in the thymus was unanticipated (Table 1; Figure 4). It was equally unexpected that we observed an ~3-fold increase in total CD4SP cells within the total T cell population. Why did PTIP576 expression only perturb the development of CD4SP unstimulated thymocytes but not CD8SP nor CD4CD8 DP cells? Our data further suggested that PTIP576 exclusively targeted the transition from CD4CD8 DP to CD4SP.

Apoptosis plays a crucial role in this transition by eliminating DP cells that do not receive the proper TCR signaling during positive selection (reviewed in [32]). This ensures that only the cells with appropriate self-antigen recognition survive and differentiate into mature CD4SP T cells. Is it possible that PTIP576 may catalyze T cell-specific apoptosis?

The primary factor that blocks thymocyte production in naive CD4 T cells is the cytokine IL-7 [33]. IL-7 acts as a crucial survival signal to prevent these cells from undergoing programmed cell death by maintaining their anti-apoptotic pathways [34]. IL-7 is constantly produced in the bone marrow and thymus to provide a continuous survival signal to naive T cells to ensure their population is maintained [33].

It will be informative to measure IL-7 levels both in naive CD4 SP thymocytes but also following antigen-specific selection *in vivo*. The latter approach will require immunization of our transgenic PTIP576-overexpressing mice with antigen + major histocompatibility antigen class 2 (MHC-11) or by polyclonal stimulation, prior to measuring expression levels of IL-7.

Finally, what underlies the unanticipated expression of our transgene in T cells? Conventional PTIP promotes homologous recombination (HR) in B cells via recruitment to double-strand break sites through its BRCT domains, which facilitates the repair of DNA damage [10]. Is it possible that this HR function could occur so that cells other than B cells (eg, in the present circumstance, T cells) could express a [BRCT1/2/3/4+ BRCT5/6−] type of PTIP?

### The predicted function and tertiary structure of the Exon (E)12–14 C-terminal region

**Figure 5.**
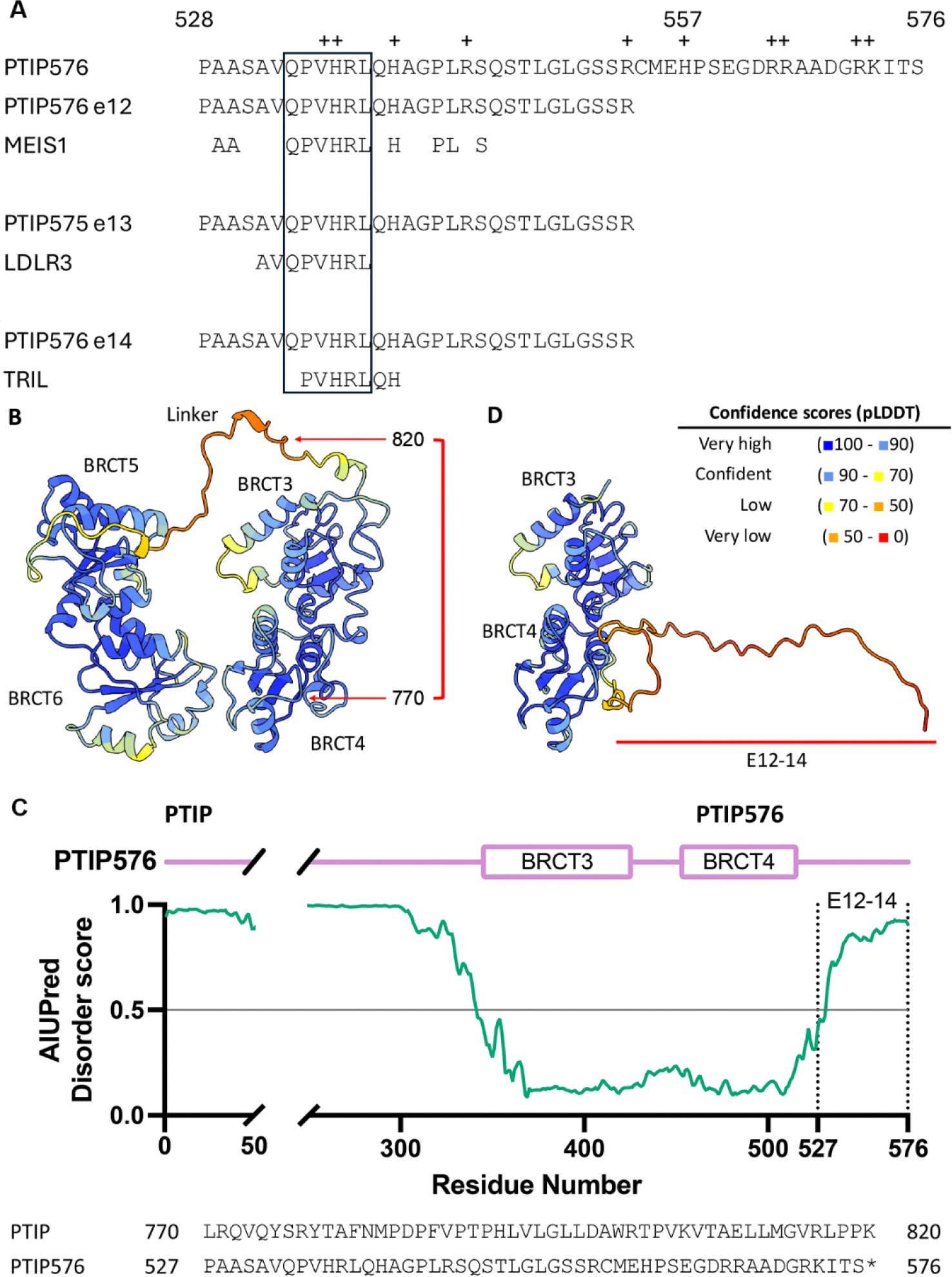
Structural modeling comparison of PTIP and PTIP576. (**A**) Alignments of hexameric repeats within Exons 12–14 of the carboxyl terminus of PTIP576. Top line: Basic residues (+) within exons (e) 12–14; 2^nd^ line: N-terminal e12 sequence successfully employed to derive homologies via data base; alignments below: exon 12 is aligned with 3 candidates that share identity (boxed): Gln-Pro-Val-His-Arg-Leu (QPVHRL) which derive from MEIS (Myeloid Ecotropic viral Integration Site 1), [35]; the LDLR (Low-Density Lipoprotein Receptor) [36]; and TRIL (TLR4-Interactor with Leucine-rich repeats) [37]. (**B**) Predicted structure of PTIP for BRCT 3–6, color-coded by pLDDT scores. (**C**) Disorder score plot calculated by AIUPred. Scores above 0.5 (indicated by a gray line) represent predicted disordered region. (**D**) Predicted structure of PTIP576. Only the central BRCT domains (3 and 4) are shown, along with the frameshifted residues, corresponding to residues 770 – 820 in PTIP. Ribbon Diagram generated by Chimera X.

By virtue of the unique mechanism of frameshift and alternative pre-mRNA splicing (Figures 1 and 2), a unique sequence of 50 amino acids was generated at the C-terminus of PTIP576. As shown in Figure 5A, the amino acid composition is extremely basic, as 21% of its residues are lysine, arginine, or histidine. We performed a database search of the complete 50 amino acid E12–14 and identified no candidates (data not shown). However, employing as query the first 30 amino acids of E12–14 (which constitutes its entire exon 12), we identified three alignments: MEIS (Myeloid Ecotropic viral Integration Site 1), a homeobox (HOX) transcription factor that regulates a number of cellular processes [35]; the LDLR (Low-Density Lipoprotein Receptor) which is primarily located on the cell membrane [36]; and TRIL (TLR4-Interactor with Leucine-rich repeats) which is involved in signaling pathways via interaction with TLR4 in the context of inflammation and immune response [37].

What draws significance from these otherwise disparate functionalities is the oligopeptide sequence Gln-Pro-Val-His-Arg-Leu (boxed in Figure 5A). A literature search has revealed no such additional homologies to date. Plus, several studies have shown that transfer into the nucleus often require “carrier proteins” [38].

To visualize the predicted three-dimensional structure of PTIP576 with the C-terminal 50 amino acids of E12–14, AlphaFold3 was used [39], and the structure was colored by predicted local distance difference test (pLDDT) scores using ChimeraX [40].

As shown in Figure 5B, the BRCT domain pairs are predicted with high confidence, connected by a large, flexible linker between BRCT3/4 and BRCT5/6. In contrast, the frameshift mutation in PTIP576 alters the amino acid sequence starting at residue 770, resulting in 50 novel residues which are not present in PTIP. Disorder scores calculated by AIUPred [41] suggest that these newly introduced residues are likely to be intrinsically disordered (Figure 5C). The frameshift occurs within the loop connecting BRCT3/4 and BRCT5/6 pairs, and the premature stop codon creates a new flexible tail (Figure 5D).

While it is difficult to predict whether this disordered region can specifically bind to any targets, its low complexity nature raises the possibility of sequestration with other PTIP576 molecules.

Thus, an important future direction is to carry out a systematic mutagenesis of resides constituting E12–14 and to design further functional assays. Alternatively, it might be prudent to employ peptides containing Pro-Val-His-Arg-Leu cores to identify E12–14-interacting proteins.

We also submit that further functional analyses of transgenic lymphocytes are required to address the intriguing hypothesis that PTIP576 derived in B cells as a loss-of-function version of conventional PTIP. In order to study both the evolution and function of PTIP576, we plan to construct transgenic knockout mice which express PTIP576 in a normal context, i.e., by directly removing the relative introns at the genomic level.

## Materials and Methods

### Cloning. PTIP576

PTIP576 was cloned from an Interleukin (IL)-2+IL-5-induced LambdaZap library constructed from a murine B cell leukemia (BCLl1). The cDNA library was constructed and described in Herrscher *et al.* [16]. We used as a probe, a fragment of IL-2 cDNA which contains 12 glutamine (CAG) repeats. The 12-CAG-containing plasmid, termed pcD-IL-2, was digested with PstI, HindIII and AceI, purified over a 7.5% SDS-PAGE gel. A 330 bp fragment containing the 12 CAG repeats was excised, extracted in 300 μl of 0.3M NaAc at 37°C and incubated overnight. The fragment was labeled by nick translation and employed to clone a 120 bp fragment from the 5’ end of PTIP576.

For library screening, ~4 ×10^6^ PFUs were plated at a density of ~1 ×10^5^/plate using *E. coli* XL1-Blue as a host. Approximately, 6.5 ml of top agar was mixed with phage and host cells, plated on NZY agar plates, and then incubated overnight at 37°C. Phage plaques on the plates were transferred to nitrocellulose filters, and duplicate filters were denatured in 2X SSC in 0.2 M Tris/HCl (pH 7.5). Filters were blotted onto Whatmann 3 mm filters and baked at ~80°C for 2 hr under vacuum. The filters were then hybridized to appropriate probes at ~1 ×10^6^ cpm/ml. Positive plaques were picked and resuspended in SM buffer (0.1 M NaCl, 10 mM MgSO_4_, 50 mM Tris-HCl and 0.01% gelatin). Eluted phage stocks were titered and replaqued for further screening until plaque purification was obtained.

### Excision of Bluescript phagemid from Lambda Zap

~200 μl of XL1-Blue cells (OD_600_~1.0) were infected with ~100 μl of phage extract (~1 × 10^9^ PFU/ml) together with 2 μl of helper phage R408 (~2 ×10^6^ PFU/ml) at 37°C for 15 min. Five ml of 2X YT broth was added and the mixture was incubated at 37°C for 6 hr with shaking, followed by heating at 70°C for 20 min. The mixture was spun for 5 min at 1000g, and the supernatant was collected. Ten μl of supernatant was added to 200 μl of XL1-Blue cells (OD_600_~1.0) and incubated at 37°C for 15 min. Phagemid-infected XL1-Blue cells were spread onto LB agar plates containing 100 μg/ml ampicillin and then incubated at 37°C overnight.

### DNA sequence analyses

Standard dideoxy/Sanger sequencing was performed on both strands of the cDNA inserts of phagemids employing sequence version 1.0 sequencing kits (USB). Confirmation was obtained by sequence of both strands using the Exoquence DNA sequencing strategy [43]; GenBank accession number PV90437.

### Southern and Northern analyses

Southern blots were performed as previously described [44]. RNAs from various tissues and cell lines described in the text were isolated with guanidinium thiocyanate followed by centrifugation over cesium chloride gradients. Approximately 10 μg of total RNA/lane was fractionated over 1.5% agarose gels containing 4% formaldehyde developed in 10 ml Phosphate Buffered Saline (PBS) and then transferred in 34 mM Disodium phosphate (DSP) and 16 mM DSP (pH 6.5). RNA was crosslinked to Genescreen (DuPont) membranes by exposure to UV light for 10 min and then baked at 80°C for 2 hr under vacuum. Membranes were prehybridized and hybridized as recommended by the manufacturer. Membranes were exposed to X-ray film for various times. Probes used for Southerns or Northerns were labeled either by nick-translation or by random priming as detailed by Sambrook *et al.* [44].

### Timed pregnancies and magnetic bead analysis of embryonic B cell progenitors

Tissue lysates (total protein) of C57BL/6 embryos from E10 to E18 were obtained from Zyagen (San Diego, CA 92127). Termination of pregnancy timing was determined by observation of vaginal plugs, with plug date considered to be day one of gestation. Embryos were collected and homogenized in protein lysis buffer supplemented with a cocktail of 7 mammalian protease inhibitors to minimize proteolysis. The extracted protein was quantified, packed in 1.5 ml tubes at a concentration of 5 mg/ml and stored at −80°C prior to use.

For embryonic analyses, we employed an EasySep^™^ Mouse Hematopoietic Progenitor Cell Isolation Kit (Stem Cells Technology; Hematopoietic progenitor cells (Catalog #19856, #18757). Briefly, cell suspensions were established by employing EasySep^™^ RapidSpheres^™^, followed by addition to the cell suspensions of EasySep^™^ Isolation Cocktail. After incubation for 2 to 5 min, the desired fraction was poured into a new tube and then pipetted equally to another set of tubes. Tubes were placed under EasySep^™^ magnets for 2.5 min and then subjected to EasyEights^™^ EasySep^™^ magnets for 5 minutes as recommended by the manufacturer. Individual tubes contained magnetically isolated CD19^−^ non-B cells, HSA^hi^/IgM^+^ immature B cells, PNA^hi^/IgD^−^ germinal center B cells as well as additional samples not employed (Figure 3A).

Immunomagnetic negative isolations were subjected to Western blotting employing a rabbit polyclonal PTIP antibody ab70434 (Abcam) generated from a synthetic peptide (database link Q6ZW49) within Human PAXIP1 which is conserved in mouse PTIP576.

### Flow cytometry

The cells were seeded and transfected as described above. At different time points after transfection, cells were washed by 2 ml PBS and trypsinized for 5 min. The cells were then washed off the plate by 5 ml of DMEM medium, and the cell suspension was transferred to a 15 ml culture tube. The cells were collected by centrifugation at 2,000 rpm for 10 minutes and the cell pellets were washed by PBS once and re-centrifuged at 2,000 rpm for 10 min. The cell pellets were resuspended in 0.5 ml of PBS for flow cytometry analysis (BD FACSCalibur Flow Cytometry System). Further description of the flow analyses is provided in the legend to Figure 5.

### Construction of PTIP576 transgenic mice

All animals were maintained in specific pathogen-free barrier facilities and were used in accordance with the IACC protocol (Protocol ID: AUP-2415–00195) approved by Institutional Animal Care and User Committee at UT Austin. C57BL breeders were purchased from The Jackson Laboratory.

To construct the transgenic vector (pEmb1-HA-mPTIP576), pEGFP-HA-PTIP576, was digested with Hind III and SmaI and the two small DNA fragments containing mPTIP576 cDNA were purified from an agarose gel, ligated into Hind III/SmaI-digested pEmb1 [18]. The resulting construct, pEmb1-PTIP576, was linearized with Not I and the DNA fragment containing PTIP576 was purified over agarose. Purified and linearized HA-PTIP576 cDNA was subsequently injected into male pronuclei of fertilized one-cell embryos of C57BL/6N mice. Breeding and maintenance of mice were performed under institutional guidelines. Mice were housed in germ-free cages and maintained with autoclaved laboratory water and chow under standard 12 hr cycles of light and dark. Among 75 mice obtained, 12 were transgenic. Eight of these transmitted the pEmb1-HA-PTIP576 into germ cells. C57Bl mouse eggs and the injected eggs were implanted into oviducts of pseudopregnant foster mothers.

### Dot blot DNA analysis of transgenic mice

Mouse tails (~1 cm in length) were digested overnight in 4 ml of SNET (10 mM Tris PH 8.0, 5 mM EDTA, 1% SDS and 400 mM NaCl) plus 60 μl proteinase K (10 mg/ml) solution in 15 ml Falcon tubes. 600 μl of digested samples were placed in a 1.5 ml Eppendorf tubes followed by adding 600 μl phenol/chloroform and vigorous vortexing for ~10 sec. The suspensions were centrifuged for 5 min and ~400 μl was removed from the top layer and placed in a 1.5 ml tube. 800 μl of cold EtOH OH was added to each 400 μl sample prior to brief vortexing and storage at −20°C. DNAs were spotted onto nitrocellulose and then crosslinked by UV light using Stratelink (Stratagene). Prior to hybridization, the probe (a purified ~300 bp fragment from EcoRI/NotI digested pEmb1HA-PTIP576) was denatured by boiling for 15 min. After the pre-hybridization solution, filters were washed 3X with gentle agitation in 2X SSC/0.5 X SET at 65°C for 15 min and three changes of wash solution of 500 ml each; the 2^nd^ wash—wash in 0.1 X SSC/0.5 X SET at 50°C for 15–20 min. After air-drying, filters were exposed via phosphoimaging for 1 hr.

### Western blot analysis of PTIP576 transgenic mice

Cell lysates from spleen, liver and thymus of transgenic mice were prepared as previously described [42]. Aliquots of ~20 μg of protein from each cell lysate were subjected to 10% SDS-PAGE. After transferring to nitrocellulose, the filters were probed with a mouse monoclonal antibody against HA (GeneScript; A01244). The antigen/antibody complexes were visualized by ECL.

### Preparation of transgenic cells for flow cytometric analysis

Bone marrow from femurs and tibias were flushed with PBS containing 0.1% sodium azide and 5% FBS (FACS media) using a 1 ml syringe and a 27G needle and then pipetted repeatedly to achieve single cell suspension. Spleens and thymuses were ground in 0.5 ml FACS media and filtered through 70–100 μm membranes to remove clumps. Cells were resuspended in FACS media and then pelleted by centrifuging for 5 min at 200g at 4°C. Each were diluted as noted above into FACS media to a 2X concentration of ~100 μl cells. After mixing and incubation on ice for ~30 min, unbound antibody (Ab) was decanted and secondary (typically Streptavidin-Fluorochrome) was diluted to 2X in FACS media, added to ~100 μl cells, mixed and incubated for 30 min on ice. Prior to FACS analysis, the cells were washed twice to remove as much unbound or non-specific bound antibody as possible.

Single-cell suspensions (~1 × 10^6^) were first stained for 20 min at 4°C with biotinylated and/or Fc-blocking Abs in flow cytometry buffer (1% [v/v] FBS and 0.1% [w/v] azide in PBS), followed by incubation with a various Abs conjugated to FITC or PE including: Anti-mouse IgM [RM121] (ab193159); anti-CD11b (M1/70; BD Pharmingen); anti-B220 (RA3–6B2; eBioscience); anti-CD138 (281–2) and anti-CD43 (S7; both from BD Pharmingen); anti-CD8 alpha [C8/144B] (ab17147; Abcam); anti-CD4 [CAL4] (ab237722; Abcam); anti-c-kit (EPR24708–25; (Abcam273119); anti-CD3 (145–2C11); PE/Cy7^®^ anti-CD25 [PC61.5] (ab210335; Abcam) and streptavidin (both from eBioscience). Data were collected on a FACSCanto2 (BD Biosciences) and were analyzed with FlowJo software (Tree Star).

## Supplementary Material

JCS-25-143-Supplmentary file

## Figures and Tables

**Table 1.** Summary of flow cytometric analyses of PTIP576 transgenic expression. Generation of the two transgenes, the C57/Bl6 mouse lines and details of mouse husbandry, the transgene vector employed [20], flow cytometry and related techniques are provided in Materials and Methods and in the legend to Figure 4. These results are representative of 3–5 independent determinations.

	Bone Marrow	Spleen	Thymus	Cell Type	
**B220+IgM+**	N	N		B cell	**Mature B cells**
**Mac-1**	N	N		Mφ	
**CD4**	N	N		T cell	
**CD8**	N	N		T cell	
**Cell numbers**	N	↑ 57%*			

**B220+cKit**	↓45%*			HSC→pre-BI	**HSC → Mature B cells**
**CD43+B220+**	↓43%*			pre-BII	
**CD25+B220+**	↑50%*			pre-BIII	
**B220+IgM+**	↑110%*			mature B	

**CD8 SP**			N		**T cells**
**CD4CD8 DP**			N		
**CD4 SP**			↑300%**		
**CD3+**	N	N	↑110%*		
**Cell numbers**			N		
