# Supplementary material for: A Newly Characterized, Two BRCT Domain-Containing Isoform of PAX-Interacting Protein (PTIP) Generated via Frame Shift and Alternative Pre-mRNA Splicing
Source: J Cell Signal. Author manuscript; Available in PMC 2026 Jan 31. (PMC12857844; doi:10.33696/signaling.6.143)
Supplement: JCS-25-143-Supplmentary file [file NIHMS2132284-supplement-JCS-25-143-Supplmentary_file.zip › Supplmentary file.pptx]

## Slide 1
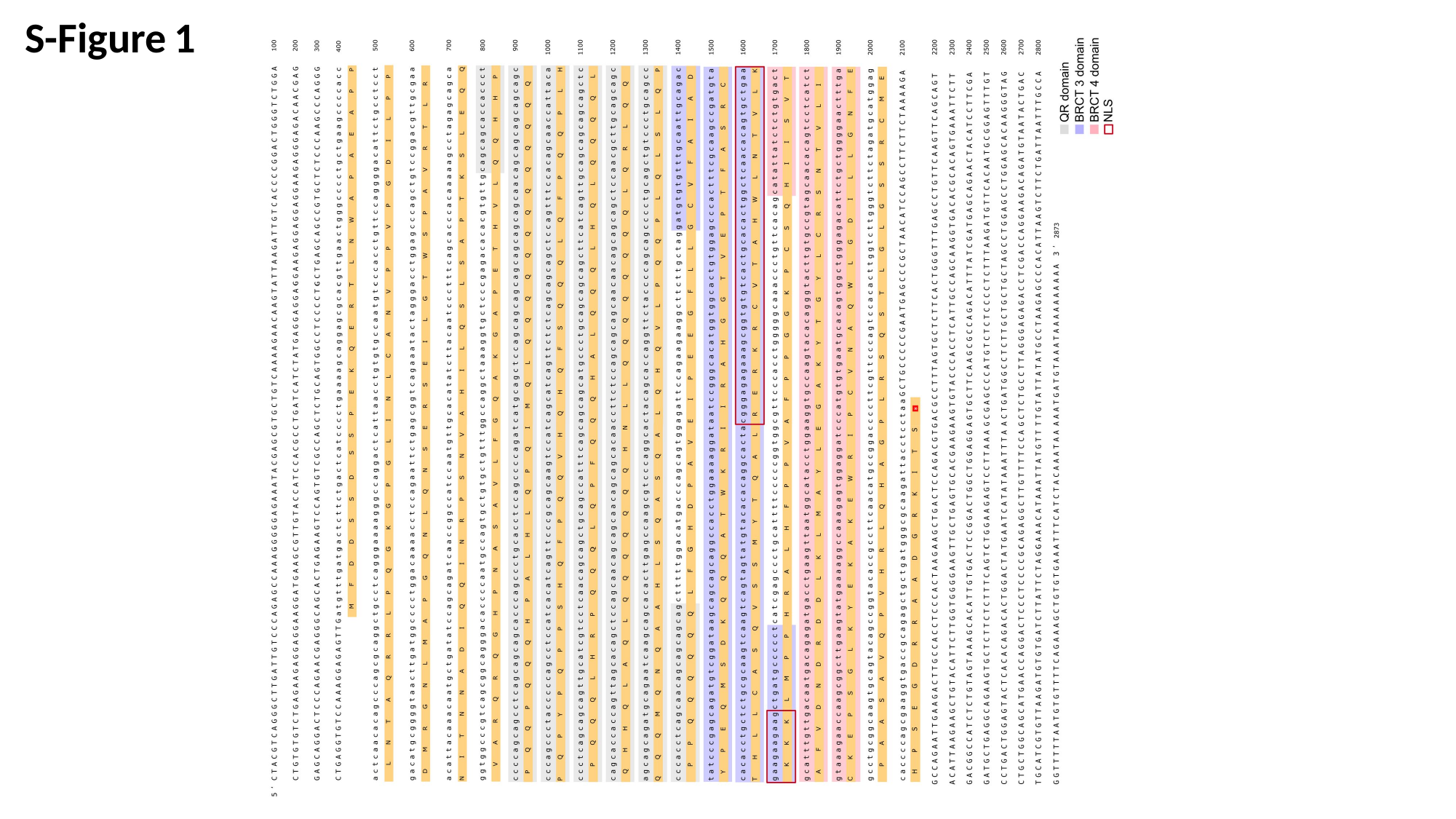

S-Figure 1

## Slide 2
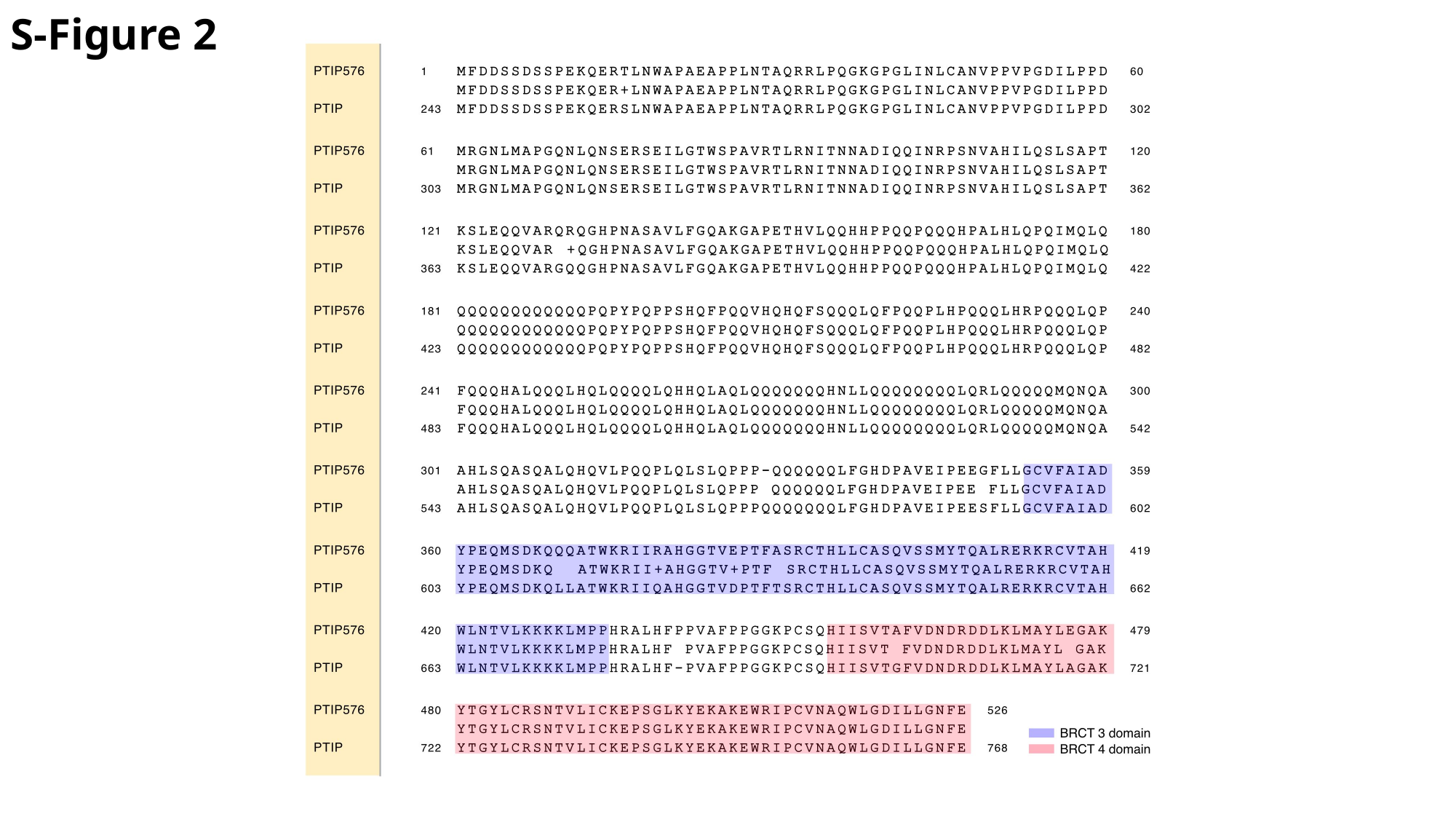

S-Figure 2

## Slide 3
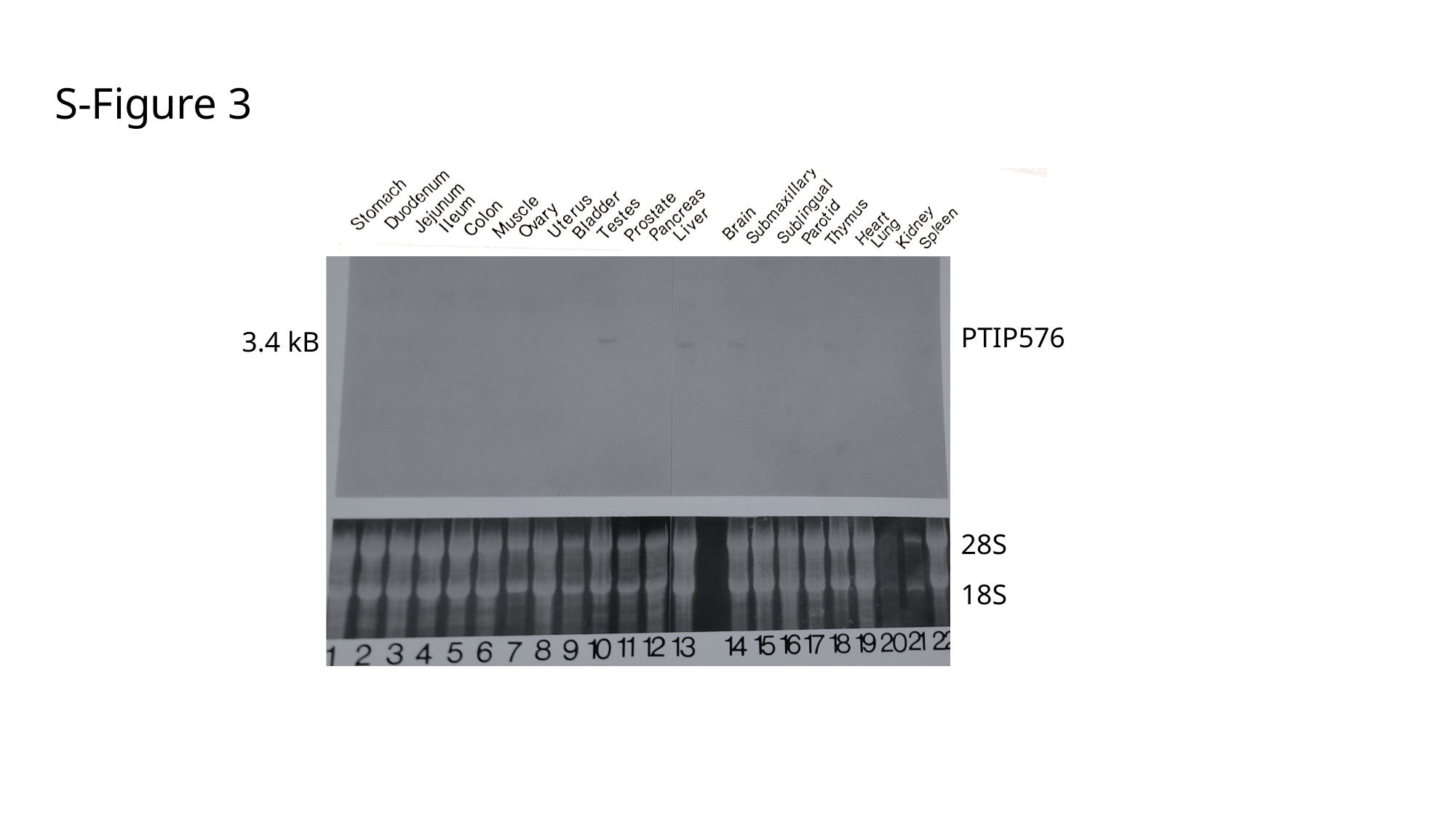

S-Figure 3
PTIP576
3.4 kB
28S
18S

## Slide 4
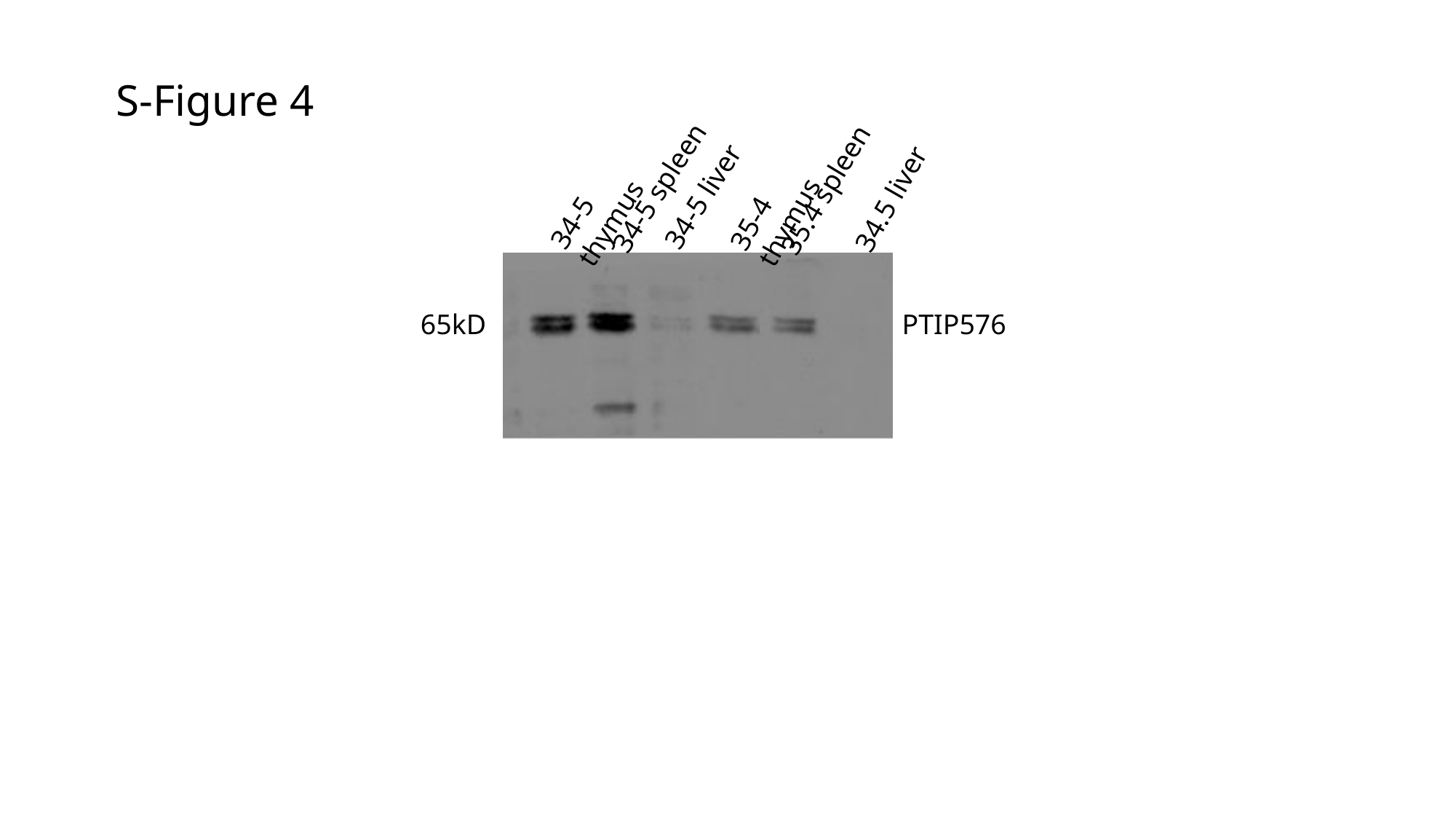

S-Figure 4
34-5 liver
35-4 thymus
34-5 thymus
34.5 liver
34-5 spleen
35.4 spleen
65kD
PTIP576
